# Human papillomavirus predicts outcome in oropharyngeal cancer in patients treated primarily with surgery or radiation therapy

**DOI:** 10.1038/sj.bjc.6605944

**Published:** 2010-10-19

**Authors:** A M Hong, T A Dobbins, C S Lee, D Jones, G B Harnett, B K Armstrong, J R Clark, C G Milross, J Kim, C J O'Brien, B R Rose

**Affiliations:** 1 Department of Radiation Oncology, Sydney Cancer Centre, Royal Prince Alfred Hospital, Building 27, Missenden Road, Camperdown, Sydney, New South Wales 2050, Australia; 2 Sydney Medical School, University of Sydney, Sydney, New South Wales, Australia; 3 Adult Cancer Program, Lowy Cancer Research Centre and Prince of Wales Clinical School, University of New South Wales, Sydney, New South Wales, Australia; 4 Discipline of Pathology, School of Medicine, University of Western Sydney, Sydney, New South Wales, Australia; 5 Department of Anatomical Pathology, Liverpool Hospital, Sydney, New South Wales, Australia; 6 Cancer Pathology, Bosch Institute, University of Sydney and Department of Anatomical Pathology, Royal Prince Alfred Hospital, Sydney, New South Wales, Australia; 7 Sydney Head and Neck Cancer Institute, Sydney Cancer Centre, Royal Prince Alfred Hospital, Sydney, New South Wales, Australia; 8 Department of Infectious Diseases and Immunology, University of Sydney, Sydney, New South Wales, Australia; 9 Pathwest Laboratory Medicine, QEII Medical Centre, Nedlands, Perth, Western Australia, Australia; 10 Sydney School of Public Health, University of Sydney, Sydney, New South Wales, Australia

**Keywords:** human papillomavirus, oropharyngeal SCC or oropharyngeal cancer, outcome, surgery, radiotherapy

## Abstract

**Objective:**

This study examines the prognostic significance of human papillomavirus (HPV) in patients with locally advanced oropharyngeal squamous cell carcinoma (SCC) treated primarily with surgery or definitive radiotherapy.

**Methods:**

One hundred and ninety-eight patients with Stage 3/4 SCC were followed up for recurrence in any form or death from any cause for between 1 and 235 months after diagnosis. HPV status was determined using HPV E6-targeted multiplex real-time PCR/p16 immunohistochemistry. Determinants of recurrence and mortality hazards were modelled using Cox's regression with censoring at follow-up dates.

**Results:**

Forty-two per cent of cancers were HPV-positive (87% type 16). HPV predicted loco-regional control, event-free survival and overall survival in multivariable analysis. Within the surgery with adjuvant radiotherapy (*n*=110), definitive radiotherapy-alone (*n*=24) and definitive radiotherapy with chemotherapy (*n*=47) groups, patients with HPV-positive cancers were one-third or less as likely to have loco-regional recurrence, an event or to die of any cause as those with HPV-negative cancers after adjusting for age, gender, tumour grade, AJCC stage and primary site. The 14 patients treated with surgery alone were considered too few for multivariable analysis.

**Conclusion:**

HPV status predicts better outcome in oropharyngeal cancer treated with surgery plus adjuvant radiotherapy as well as with definitive radiation therapy±chemotherapy.

It is now accepted that human papillomavirus (HPV) is an aetiological agent of up to 60% of oropharyngeal squamous cell carcinomas (SCCs) (Gillison *et al*, 2000; Vidal and Gillison, 2008) and there have been recent reports that the incidence of HPV-induced oropharyngeal cancer is increasing (Frisch *et al*, 2000; Hammarstedt *et al*, 2006; Hong *et al*, 2010c). Our studies and others have shown that the HPV-positive subset of cancers is biologically distinct (Andl *et al*, 1998; Li *et al*, 2003a; Weinberger *et al*, 2006; Fakhry *et al*, 2008; Vidal and Gillison, 2008; Lassen *et al*, 2009; Sedaghat *et al*, 2009; Hong *et al*, 2010a, 2010b). Most notable has been the association of HPV with a favourable clinical outcome.

Radiation therapy has an important role in the management of oropharyngeal cancers, either as definitive therapy or as an adjuvant therapy after surgery. Altered fractionation and concurrent chemotherapy have been developed to maximise disease control (Pignon *et al*, 2000; Bourhis *et al*, 2006). Recent studies have provided evidence that the favourable prognosis associated with HPV relates to a better response to radiation therapy used either alone (Lassen *et al*, 2009; Sedaghat *et al*, 2009) or in combination with chemotherapy (Kumar *et al*, 2007; Fakhry *et al*, 2008). There are, however, limited data on the relationship of HPV status to the outcome of oropharyngeal cancers treated with surgery with or without adjuvant radiation therapy (Licitra *et al*, 2006; Fischer *et al*, 2008, 2010).

There is increasing use of HPV status to guide treatment of oropharyngeal SCC (Shoushtari *et al*, 2010). This study examines the effect of HPV on outcomes after surgery with adjuvant radiation therapy or with definitive radiation therapy (with or without chemotherapy) in a large series of locally advanced oropharyngeal SCCs from the same geographic region.

## Materials and methods

### Study population

Investigations were carried out on 202 consecutive patients with AJCC Stage 3 and 4 oropharyngeal SCC treated with curative intent at hospitals in Sydney, Australia, between 1987 and 2006. One hundred and forty-six cases were from Royal Prince Alfred Hospital. Human papillomavirus status for two patients could not be determined (a 71-year-old man with a Stage 3 tonsillar SCC treated with definitive radiation therapy and a 46-year-old man with a Stage 4 oropharyngeal SCC treated with definitive radiation therapy and concurrent chemotherapy), and two patients were missing adequate follow-up data (a 46-year-old man with a Stage 4 tonsillar SCC and a 68-year-old man with Stage 4 tonsillar SCC), leaving 198 patients. The study was approved by the ethics committees of Sydney South West Area Health Service (Protocols X05-0308, CH62/6/2006-041, 2006/055). Most of the demographic and clinicopathological data were retrieved from the database of the Sydney Head and Neck Cancer Institute at Royal Prince Alfred Hospital, which spans the study period. Department of Radiation Oncology and Anatomical Pathology databases and hospital records were used to verify and input missing data as required.

Patients were followed up for the occurrence of an event, defined as recurrence in any form or death from any cause, for between 1 and 235 (median 26) months after diagnosis. Patients who did not experience an event before the end of follow-up were known at that date to be alive and free of recurrence.

### Laboratory studies

#### HPV status of cancers

Evidence that HPV is transcriptionally active or localised to the nuclei of tumour cells is needed to establish causality in head and neck cancer (Vidal and Gillison, 2008). Overexpression of p16 resulting from downregulation of retinoblastoma protein by HPV E7 has been used as a surrogate marker of HPV E7 expression in several studies (Klussmann *et al*, 2003; Licitra *et al*, 2006). In our study, an HPV-positive cancer was defined as one testing positive for both HPV DNA and p16 (Smeets *et al*, 2007). The presence and type of HPV DNA were determined on two to six 4–5 *μ*m sections of formalin-fixed paraffin-embedded cancer, using an HPV E6-based multiplex real-time PCR assay (MT-PCR) modified from the method of Stanley and Szewczuk (2005). This assay simultaneously detects and identifies 21 HPV types (16, 18, 31, 33, 35, 39, 45, 51, 52, 56, 58, 59, 66, 68, 70, 73, 82, 53, 6, 11 and 26). The HPV 16 assay (Supplementary Figure 1) has a sensitivity of 400 viral genome copies per ml at the 95% confidence level (CI) and compares well with our nested PCR method, which was validated against previously published PCR methods (Brestovac *et al*, 2005). DNA was extracted using the RNA QIAmp RNA viral mini kit (Qiagen, Hilden, Germany). The first step involved incorporation of all primer pairs into two PCR mixes and amplification for 20 cycles in a conventional thermocycler. Products from each of these mixes were then passed into triplex TaqMan real-time PCR assays with probes having FAM, VIC or Cy5 labels and cycling performed for 40 cycles in a real-time thermocycler (RotorGene 6000, Corbett Research, Mortlake, NSW, Australia). A Corbett CAS 1200 robot was used to inoculate samples and to make the transfers from the initial PCR into the TaqMan PCR mixes. A measured amount of equine herpesvirus was introduced into the extraction lysis buffer and used to monitor the efficiency of DNA extraction and removal of PCR inhibitors. Stringent precautions were used to minimise the possibility of cross-contamination. Section cutting was carried out in an institution remote from that used for the PCR analyses; microtome blades were cleaned with xylene between blocks, and changed frequently. PCR procedures were carried out in a laboratory equipped with separate rooms for preparing reagents, extracting samples, handling PCR products and for thermocycling. Water blanks were included after every fifth sample. The presence of cancer cells in sections used for HPV testing was confirmed by a pathologist (CSL) in an H&E-stained section cut after those for HPV analysis. All discrepant HPV DNA/p16 results were confirmed by retesting for both HPV DNA and p16. No attempt was made to microdissect cancer from surrounding tissue.

Expression of p16 was carried out by semiquantitative immunohistochemistry. Antigen retrieval was carried out using Target Retrieval Solution pH9 (Dako Corporation, Carpinteria, CA, USA) in a microwave oven for 10 min on high setting. After cooling, slides were placed in an autostainer (Dako). Endogenous peroxidase was blocked using 0.3% hydrogen peroxide in Tris-buffered saline (TBS); sections were incubated with primary antibody (Clone JC2 Neomarkers, Fremont, CA, USA) (one out of 200) for 30 min, washed in TBS, treated with the EnVision Flex Dual Link horseradish peroxidase/DAB visualisation system (Dako) and then counterstained with haematoxylin. Staining was evaluated by three observers, including at least one pathologist (CSL). Typically, strong diffuse p16 staining was seen in the nucleus and cytoplasm of cancer cells with the proportion essentially all or none (Supplementary Figures 2a and 2b). Weak focal staining was recorded as negative. All researchers were blinded to clinical and other laboratory data until results were finalised. The grade for all cancers was reviewed by two study pathologists (CSL, JRK). Any variation from a previous report or between observers was resolved over a double-headed microscope.

### Statistical analyses

Associations between HPV status and clinicopathological characteristics were assessed using a two-sample *t*-test for the continuous variable age and chi-squared tests for categorical variables. One-way analysis of variance and chi-squared tests were used to compare patient characteristics in four treatment groups: surgery alone; surgery with adjuvant radiation therapy; definitive radiation therapy alone; and definitive radiation therapy with chemotherapy.

Survival analyses were conducted for the outcomes of loco-regional recurrence, event-free survival and overall survival, with time to each outcome calculated from the date of diagnosis. An event was defined as recurrence in any form or death from any cause, with only the first event taken into account. Patients without events were censored at the date of last follow-up. Univariate associations between HPV status and time to loco-regional recurrence, an event or death from any cause, were summarised for all patients and separately for each treatment group using Cox's proportional hazards models. Unadjusted survival curves were obtained using Kaplan–Meier estimates.

Overall multivariable survival models were constructed with inclusion of HPV status and other known or potential correlates of outcome: age, gender, tumour grade, AJCC stage, primary site and treatment modality. Multivariable survival models were constructed separately for the three radiation therapy treatment groups, adjusting for HPV status, age, gender, tumour grade, AJCC stage and primary site where possible. Multivariable analyses were not carried out for the surgery-alone group because of the small numbers (14).

We assessed whether the effect of HPV differed by treatment type by using interaction terms between HPV status and treatment (categorised as surgery with adjuvant radiation therapy, definitive radiation therapy with chemotherapy and definitive radiation therapy alone), and adjusting only for age, tumour grade, tumour stage and site to avoid potential overfitting problems owing to small numbers. Adjusted survival curves by HPV status and treatment were obtained for the three treatment groups using Cox regression models, again adjusting only for age, tumour grade, stage and site. All analyses were conducted using the SAS System for Windows (SAS Institute, Cary, NC, USA) and Stata Statistical Software (Stata Corporation, College Station, TX, USA).

## Results

### HPV status and type distribution

Forty-two per cent (83 out of 198) of the cancers were HPV-positive. Human papillomavirus type 16 accounted for 87% (72 out of 83) of the HPV-positive cases. Human papillomavirus types 35 and 18 were the most common other HPV types. Five cancers positive for HPV type 16 also contained a second HPV type (two 35, one each of 33, 39 and 56). Twenty-one (11%) cancers tested HPV DNA positive/p16 negative and three (2%) cancers were HPV DNA negative/p16 positive (Supplementary Table 1). The 21 HPV DNA-positive/p16-negative samples were included in the HPV-negative group, as the molecular and phenotypic characteristics of this group resemble those of HPV-negative/p16-negative cancers (our unpublished data, Smeets *et al*, 2007; Weinberger *et al*, 2009). The three patients with HPV-negative/p16-positive cancers were excluded from further analyses because it was not known whether they were induced by an HPV type undetectable by the assay or were HPV negative (p16 upregulated through an HPV-unrelated pathway). They were too few to analyse separately. Thus, the total number of cancers in the final analysis was 195.

The patients’ characteristics are summarised in Table 1. Compared with patients with HPV-negative cancers, patients with HPV-positive cancers were younger (mean age 54.4 *vs* 62.6 years, *P*<0.0001), more likely to have primary disease in the tonsil (*P*=0.003) and had higher grade cancers (*P*=0.002). Patients with HPV-positive cancers were also more likely to have Stage 4 than Stage 3 cancers (*P*=0.03), and this was driven by more advanced nodal disease (*P*=0.02).

### Treatment

The choice of treatment modality was based primarily on clinician and institution preference. Of the 195 patients analysed, 14 were treated with radical surgery alone (five declined adjuvant radiation therapy, one died of other than head and neck cancer before adjuvant radiation therapy and the remaining eight were not referred for adjuvant radiation therapy for unknown reasons). One hundred and ten patients were treated with surgery with adjuvant radiation therapy (five with concurrent chemotherapy and five with induction chemotherapy) (Table 1). Fourteen of the 17 patients with T3N0 tumours treated with surgery and adjuvant radiotherapy had elective neck dissections. Seventy-one patients were treated with definitive radiation therapy. Of these, 47 had chemoradiotherapy (35 concurrent, 12 induction) and 24 had radiation alone. Within the surgery with adjuvant radiotherapy group, separate analysis of the 10 patients who received chemotherapy was not carried out, and within the definitive radiotherapy group, patients receiving concurrent radiotherapy were grouped with those having induction chemotherapy because of small numbers.

The 35 patients who received concurrent chemotherapy and definitive radiation therapy (mainly 5-fluorouracil or 3-weekly cisplatin) were treated between 1990 and 2006; all but three of these patients were treated after 1996. These 35 patients were equally divided between the HPV-positive and -negative groups. Over the study period, there was a change in definitive radiation dose scheduling from 60 Gy in 30 fractions over 6 weeks in the early 1990s to 70 Gy in 35 fractions over 7 weeks from 1999. The dose of adjuvant radiation therapy also increased over the study period from 50 Gy in 25 fractions to 60 Gy in 30 fractions in more recent years. All patients were treated with multiple photon fields using standard fractionation. Patients receiving surgery alone were older than other patients and patients receiving definitive radiotherapy with chemotherapy were more likely to have tumours located in the base of tongue (Table 2).

### Outcome analyses

#### Effect of HPV status

One patient was missing loco-regional recurrence status, yielding 194 patients for analysis. Loco-regional recurrence occurred in 54 (28%) of 194 patients: in four of the 14 (29%) patients treated with surgery alone, 24 of the 109 (22%) patients treated with surgery and adjuvant radiation therapy, 10 of the 25 (40%) patients treated with definitive radiotherapy alone and 16 of the 47 (34%) patients treated with definitive radiation therapy with chemotherapy. Recurrence occurred at the primary site in 30 patients and in the regional nodal area (with disease controlled at the primary site) in 24 patients. Sixteen patients developed distant metastasis as the first site of recurrence, none of whom had simultaneous loco-regional recurrence.

Univariate analysis of data from all patients showed that HPV-positive cancers were less likely to recur loco-regionally than were HPV-negative cancers (Table 3). This association remained after adjustment for age, gender, grade, stage, primary site within the oropharynx and treatment group (hazard ratio (HR)=0.27; 95% CI: 0.13–0.52; Table 3).

**Figure 1:**
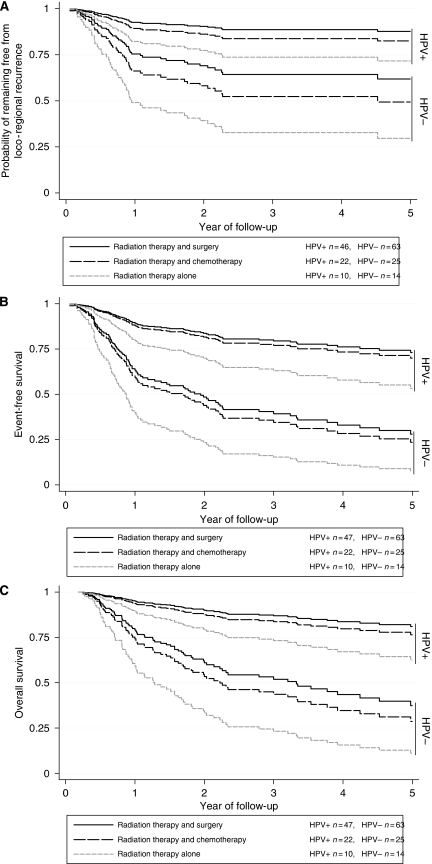
Probability of (**A**) remaining free from loco-regional recurrence, (**B**) survival free from an event and (**C**) overall survival by HPV status and type of radiation therapy, adjusted for age, tumour grade, stage and site.

There was little evidence that the effect of HPV on loco-regional recurrence differed by treatment group (Figure 1A); *P*=0.7 for interaction between HPV status and treatment group in the adjusted analysis. After adjustment for age, tumour grade and tumour stage, the risk of loco-regional recurrence associated with HPV-positive cancers relative to HPV-negative cancers was about one-third (HR=0.30; 95% CI: 0.10–0.82) in the surgery with adjuvant radiation therapy group, one-eighth (HR=0.12; 95% CI: 0.01–0.61) in the definitive radiation therapy-alone group and one-quarter (HR=0.25; 95% CI: 0.06–0.79) in the definitive radiation therapy with chemotherapy group (Table 3).

There were 99 events involving nine of the 14 (64%) patients treated with surgery alone, 48 of the 110 patients (44%) treated with surgery and adjuvant radiation therapy, 16 of the 24 (67%) patients treated with definitive radiotherapy alone and 26 of the 47 (55%) patients treated with definitive radiation therapy and chemotherapy. Univariate analysis of data from all patients showed that patients with HPV-positive cancers were less likely to suffer an event than those with HPV-negative cancers (Table 3). This association remained after adjustment for age, gender, grade, stage, primary site within the oropharynx and treatment type (HR=0.23; 95% CI: 0.14–0.39).

There was little evidence that the effect of HPV on event-free survival differed by treatment group (Figure 1B); *P*=0.4 for interaction between HPV status and treatment group in the adjusted analysis. After adjustment for age, tumour grade and stage, the risk of an event associated with HPV-positive cancers relative to HPV-negative cancers was about one fifth (HR=0.19; 95% CI: 0.08–0.40) in the surgery with adjuvant radiation therapy group, about one-seventh (HR=0.14; 95% CI: 0.02–0.54) in the definitive radiation therapy-alone group and about one-third (HR=0.36; 95% CI: 0.14–0.85) in the definitive radiation therapy with chemotherapy group (Table 3).

There were 82 deaths from any cause involving seven of the 14 patients (50%) treated with surgery alone, 38 of the 110 patients (35%) receiving surgery with adjuvant radiation therapy, 13 of the 24 patients (54%) receiving definitive radiotherapy alone and 24 of the 47 patients (51%) receiving definitive radiation therapy with chemotherapy. Univariate analysis of data from all patients showed that patients with HPV-positive cancers were less likely to die of any cause than those with HPV-negative cancers (Table 3). This association remained after adjustment for age, gender, grade, stage, primary site within the oropharynx and treatment type (HR=0.24, 95% CI: 0.13–0.42).

There was little evidence that the effect of HPV on overall survival differed by treatment group (Figure 1C); *P*=0.3 for interaction between HPV status and treatment group in the adjusted analysis. After adjustment for age, tumour grade and stage, the risk of death from any cause associated with HPV-positive cancers relative to HPV-negative cancers was almost one-tenth (HR=0.11; 95% CI: 0.04–0.28) in the surgery with adjuvant radiation therapy group, almost one-tenth (HR=0.11; 95% CI: 0.01–0.52) in the definitive radiation therapy-alone group and about one-third (HR=0.37; 95% CI: 0.13–0.90) in the definitive radiation therapy with chemotherapy group (Table 3).

#### Effects of clinical variables

On the basis of multivariable analyses of all patients adjusting for HPV status, type of treatment was the only clinical characteristic that showed evidence of an association with loco-regional recurrence (*P*=0.06), with the surgery and adjuvant radiotherapy group having the lowest risk of loco-regional recurrence and the surgery-only group having the highest risk.

Multivariable predictors of event-free survival were identified as stage (*P*=0.0001), site of cancer (*P*=0.04) and type of treatment (*P*=0.0003) after adjusting for HPV status. Poorer outcomes were seen in Stage 4 patients, patients with cancer in sites other than tonsil and patients receiving surgery only.

After adjusting for HPV status, clinical characteristics that showed evidence of associations with overall survival were identified as stage (*P*<0.0001), site of cancer (*P*=0.001) and type of treatment (*P*=0.05). Again, poorest outcomes were seen in Stage 4 patients, patients with cancer in sites other than tonsil and patients receiving surgery only.

When stratified by HPV status, outcome-adjusted survival against each end point was consistently highest for surgery with adjuvant radiotherapy, intermediate for definitive radiotherapy with chemotherapy and lowest for definitive radiotherapy alone (Figure 1A–C). (Patients treated with surgery alone were too few to include in these comparisons.) These differences in outcome by treatment type should be interpreted with caution because they are not randomised comparisons and may be affected by confounding by indications for particular treatment types. For each outcome, there was little evidence that the effect of treatment type was modified by stage or by site of cancer (*P* for interaction >0.3 in each case).

## Discussion

Recent studies have suggested that the favourable outcome associated with HPV in oropharyngeal cancer is owing to an increased sensitivity of virus-related cancers to radiation therapy or chemoradiotherapy (Fakhry *et al*, 2008; Kumar *et al*, 2008; Lassen *et al*, 2009; Sedaghat *et al*, 2009). Data from patients treated primarily with surgery (with or without adjuvant radiotherapy) are limited, but there have been recent reports of a survival advantage for those with HPV-positive cancers (Licitra *et al*, 2006; Fischer *et al*, 2010). Our findings show that HPV status is a strong predictor of loco-regional recurrence and survival in patients treated either by surgery with adjuvant radiotherapy or by an organ-preserving approach using definitive radiotherapy with or without chemotherapy. Mounting evidence that the effect of HPV on outcome in oropharyngeal cancer is independent of treatment modality is not inconsistent with theories that HPV-positive cancers are more radiosensitive than HPV-negative cancers, but rather suggests that other factors including immune surveillance to virus-specific tumour antigens and a lack of field cancerisation (Mellin *et al*, 2000; Lindel *et al*, 2001) may have an important contributing role.

As most recent studies have focused on HPV and outcome following definitive radiotherapy or chemoradiotherapy, the interest of this study centres on patients with surgery as primary treatment. Our results suggest that outcomes in patients with locally advanced HPV-positive cancers treated with surgery and adjuvant radiotherapy were as good as, and possibly better than, those treated with definitive radiotherapy with or without chemotherapy. This also appeared to be the case for HPV-negative cancers, and the effect of HPV status on outcome did not appear to differ materially between these treatment groups. In their study of patients with all stages of disease, Licitra *et al* (2006) reported an effect of HPV on outcome in patients treated with surgery alone as well as those receiving surgery with adjuvant radiotherapy. In our study, overall outcomes were poorer in patients treated with surgery alone than in other treatment groups and there was only weak evidence for a better outcome for HPV-positive cancers treated with surgery alone on univariate analysis. However, we only included patients with locally advanced disease who, in our centre, have long been routinely referred for adjuvant radiotherapy (six of the 14 patients did not have adjuvant radiation therapy as recommended). The numbers receiving surgery only were too few to adequately evaluate outcome by HPV status. It was, however, worth noting that this group of patients tended to be older and to have HPV-negative cancer.

The seemingly lower HRs for outcome of HPV-positive cancer relative to HPV-negative cancer in the definitive radiotherapy-only group than in the definitive radiotherapy with chemotherapy group were unexpected. However, the wide CIs about the HRs and the high *P*-values for interaction make any inference from this difference very uncertain. Moreover, we cannot exclude selection bias relating to performance status, co-morbidities, smoking and alcohol exposure that might affect the HRs (Hafkamp *et al*, 2008; Ang *et al*, 2010; Maxwell *et al*, 2010).

The heterogeneity of disease within the head and neck region is well recognised. Although oropharyngeal SCCs are regarded as a relatively homogeneous group, studies with the statistical power to examine site within the oropharynx have found that patients with base of tongue SCCs have the worst prognosis (Zhen *et al*, 2004; Sundaram *et al*, 2005). Our overall findings indicate that site within the oropharynx is an independent risk factor for survival with tonsillar cancers having the best outcome. This finding suggests that stratification of future trials of oropharyngeal cancer by subsite may be worthwhile.

The proportion of oropharyngeal cancers attributable to HPV has varied from 19% to more than 60% across different studies (Gillison *et al*, 2000; Vidal and Gillison, 2008). Our overall HPV positivity rate of 46% is lower than that reported in some recent studies, but this is explained by the 20-year period of the study. By 2005–2006, rates in our centre had risen to 66% (Hong *et al*, 2010c). This increase is consistent with trends in other western countries (Frisch *et al*, 2000; Hammarstedt *et al*, 2006; Chaturvedi *et al*, 2008). Variation in HPV-positivity rates across different studies is also attributable to differences in the specificity and sensitivity of the HPV detection assays. However, there do seem to be geographic or ethnic differences (Li *et al*, 2003b, 2007), which highlights the need for standardised procedures for determining HPV status (Braakhuis *et al*, 2009). The proportion of our cancers testing HPV DNA positive/p16 negative, indicating that the virus is not causal, is lower than that in some studies (van Houten *et al*, 2001; Wiest *et al*, 2002; Weinberger *et al*, 2006), despite the high sensitivity of our HPV DNA assay.

Testing for HPV status of oropharyngeal SCCs is increasing in clinical practice, but there is no level 1 or 2 evidence to guide treatment based on HPV status. We conclude that patients with HPV-positive locally advanced oropharyngeal SCC have a more favourable prognosis than HPV-negative cancers, regardless of whether they are treated with radical surgery plus adjuvant radiation therapy or by an organ-preserving approach using definitive radiation therapy with or without chemotherapy. The possibility that type of treatment, as well as HPV status, may influence outcome warrants investigation in randomised controlled trials.

## Figures and Tables

**Table 1:** Demographic and clinical characteristics of the study population

	**All patients (*n*=195)**	**HPV positive (*n*=83)**	**HPV negative (*n*=112)**	**HPV-positive *vs* HPV-negative *P*-value** a
Mean age at diagnosis (range)	59.1 (range 34–84)	54.4 (range 34–84)	62.6 (range 44–84)	<0.0001
				
*Gender*				
Males	159 (82%)	69 (83%)	90 (80%)	0.6
Females	36 (18%)	14 (17%)	22 (20%)	
				
*Location*				
Tonsil	124 (64%)	61 (73%)	63 (56%)	0.003
Base of tongue	36 (18%)	16 (19%)	20 (18%)	
Other subsites	35 (18%)	6 (7%)	29 (26%)	
				
*T classification* b				
T1	21 (11%)	13 (16%)	8 (7%)	0.03
T2	60 (31%)	31 (37%)	29 (26%)	
T3	72 (37%)	23 (28%)	49 (44%)	
T4	41 (21%)	16 (19%)	25 (23%)	
				
*N classification* b				
N0	45 (23%)	12 (14%)	33 (30%)	0.02
N1	56 (29%)	21 (25%)	35 (32%)	
N2	74 (38%)	39 (47%)	35 (32%)	
N3	19 (10%)	11 (13%)	8 (7%)	
				
*Stage*				
3	78 (40%)	26 (31%)	52 (46%)	0.03
4	117 (60%)	57 (69%)	60 (54%)	
				
*Grade*				
1, 2	118 (61%)	40 (48%)	78 (70%)	0.002
3	77 (39%)	43 (52%)	34 (30%)	
				
*Treatment*				
Surgery alone^c^	14 (7%)	4 (5%)	10 (9%)	0.7
Surgery and adjuvant RT^d^	110 (56%)	47 (57%)	63 (56%)	
Definitive RT alone	24 (12%)	10 (12%)	14 (13%)	
Definitive RT and CT^e^	47 (24%)	22 (27%)	25 (22%)	

Abbreviations: HPV=human papillomavirus; RT=radiation therapy; CT=chemotherapy.

a Test for heterogeneity.

b One missing observation.

c Includes one patient who also received induction CT.

d Includes 10 patients who also received CT (five induction and five concurrent).

e Induction CT (*n*=12) and concurrent CT (*n*=35).

**Table 2:** Characteristics of patients by treatment group

	**Surgery alone (*n*=14)**	**Surgery with adjuvant RT (*n*=110)**	**Definitive RT alone (*n*=24)**	**Definitive RT with CT (*n*=47)**	***P*-value** a
Mean age at diagnosis (range)	67.1 (44–84)	58.2 (34–84)	60.7 (38–84)	58.2 (38–83)	0.03
					
*Gender*					
Males	10 (71%)	85 (77%)	22 (92%)	42 (89%)	0.1
Females	4 (29%)	25 (23%)	2 (8%)	5 (11%)	
					
*Location*					
Tonsil	9 (65%)	79 (72%)	16 (67%)	20 (43%)	0.02
Base of tongue	2 (14%)	16 (15%)	2 (8%)	16 (34%)	
Other subsites	3 (21%)	15 (14%)	6 (25%)	11 (23%)	
					
*Stage*					
3	8 (57%)	39 (35%)	9 (38%)	22 (47%)	0.3
4	6 (43%)	71 (65%)	15 (63%)	25 (53%)	
					
*Grade*					
1, 2	10 (71%)	62 (56%)	15 (63%)	31 (66%)	0.5
3	4 (29%)	48 (44%)	9 (38%)	16 (34%)	
					
*HPV status*					
Negative	10 (71%)	63 (57%)	14 (58%)	25 (53%)	0.7
Positive	4 (29%)	47 (43%)	10 (42%)	22 (47%)	

Abbreviations: CT=chemotherapy; HPV=human papillomavirus; RT=radiation therapy.

a Test for heterogeneity.

**Table 3:** Associations between HPV status and risk of recurrence, event and/or death

**Treatment group**										
	**All (*n*=195)**		**Surgery alone (*n*=14)**		**Surgery with adjuvant radiotherapy (*n*=110)**		**Definitive radiotherapy alone (*n*=24)**		**Definitive radiotherapy and chemotherapy (*n*=47)**	
	**HR (95% CI)**	***P*-value**	**HR (95% CI)**	***P*-value**	**HR (95% CI)**	***P*-value**	**HR (95% CI)**	***P*-value**	**HR (95% CI)**	***P*-value**
*Loco-regional recurrence*										
*Univariate*										
*HPV*										
Negative	1.0		1.0		1.0		1.0		1.0	
Positive	0.34 (0.17, 0.61)	0.0002	0.71 (0.04, 5.57)	0.8	0.38 (0.14, 0.91)	0.03	0.18 (0.03, 0.75)	0.02	0.36 (0.10, 1.03)	0.06
*Adjusted*										
*HPV*										
Negative	1.0				1.0		1.0		1.0	
Positive	0.27 (0.13, 0.52)^a^	<0.0001	Not estimated^b^		0.30 (0.10, 0.82)^c^	0.02	0.12 (0.01, 0.61)^d^	0.009	0.25 (0.06, 0.79)^c^	0.02
										
*Event-free survival*										
*Univariate*										
*HPV*										
Negative	1.0		1.0		1.0		1.0		1.0	
Positive	0.31 (0.19, 0.49)	<0.0001	0.50 (0.07, 2.10)	0.4	0.28 (0.13, 0.53)	<0.0001	0.19 (0.04, 0.60)	0.004	0.46 (0.19, 1.03)	0.06
*Adjusted*										
*HPV*										
Negative	1.0				1.0		1.0		1.0	
Positive	0.23 (0.14, 0.39)^a^	<0.0001	Not estimated^b^		0.19 (0.08, 0.40)^c^	<0.0001	0.14 (0.02, 0.54)^d^	0.003	0.36 (0.14, 0.85)^c^	0.02
										
*Overall survival*										
*Univariate*										
*HPV*										
Negative	1.0		1.0		1.0		1.0		1.0	
Positive	0.29 (0.17, 0.48)	<0.0001	0.98 (0.14, 4.57)	1.0	0.18 (0.07, 0.40)	<0.0001	0.24 (0.05, 0.82)	0.02	0.43 (0.17, 0.99)	0.05
*Adjusted*										
*HPV*										
Negative	1.0				1.0		1.0		1.0	
Positive	0.24 (0.13, 0.42)^a^	<0.0001	Not estimated^b^		0.11 (0.04, 0.28)^c^	<0.0001	0.11 (0.01, 0.52)^d^	0.004	0.37 (0.13, 0.90)^c^	0.03

Abbreviations: HPV=human papillomavirus; HR=hazard ratio; CI=confidence interval; RT=radiation therapy; CT=chemotherapy.

a Adjusted for age, gender, tumour grade, tumour stage, primary site and treatment.

b Multivariable analyses not carried out owing to small numbers.

c Adjusted for age, gender, tumour grade, tumour stage and primary site.

d Adjusted for age, tumour grade and tumour stage; gender and primary site not adjusted owing to categories with low numbers of events.
