# Supplementary material for: Human papillomavirus predicts outcome in oropharyngeal cancer in patients treated primarily with surgery or radiation therapy
Source: Br J Cancer. 2010 Oct 19;103(10):1510–7. doi: 10.1038/sj.bjc.6605944 (PMC2990586; doi:10.1038/sj.bjc.6605944)
Supplement: Supplementary Information [file 6605944x1.doc]

Supplementary Figure 1

HPV16 E6 PCR real-time curves generated in the RotorGene 6000 thermocycler from some samples included in this study.

Supplementary Figure 2

1. typical p16 staining for an HPV DNA positive cancer.
2. typical p16 staining for an HPV DNA negative cancer.

Supplementary Table 1

Relationship between HPV DNA and p16 from 198 tumor samples.

Supplementary Figure 1

HPV16 E6 PCR real-time curves generated in the RotorGene 6000 thermocycler from some samples included in this study.


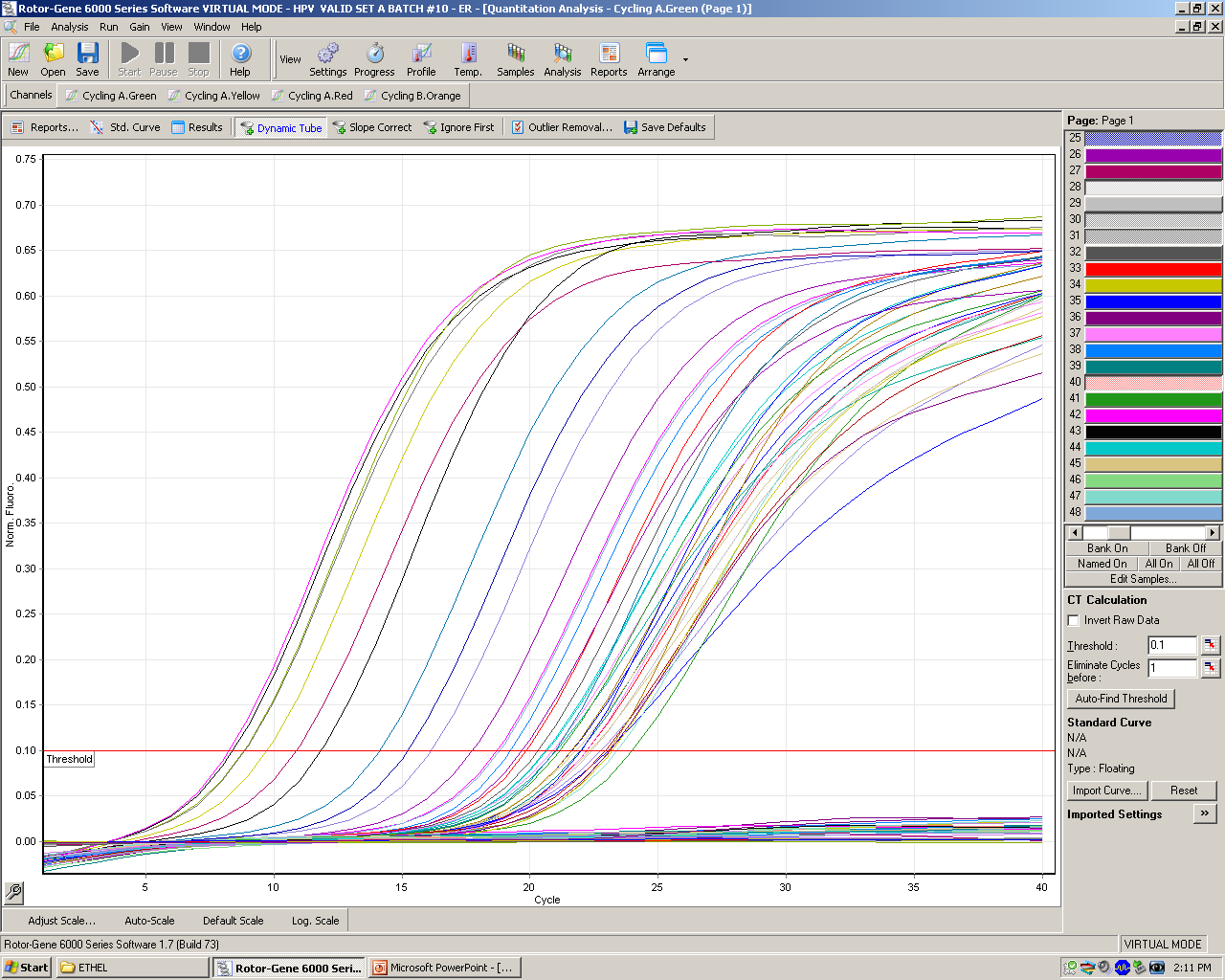


Supplementary Figure 2

1. typical p16 staining for an HPV DNA-positive cancer


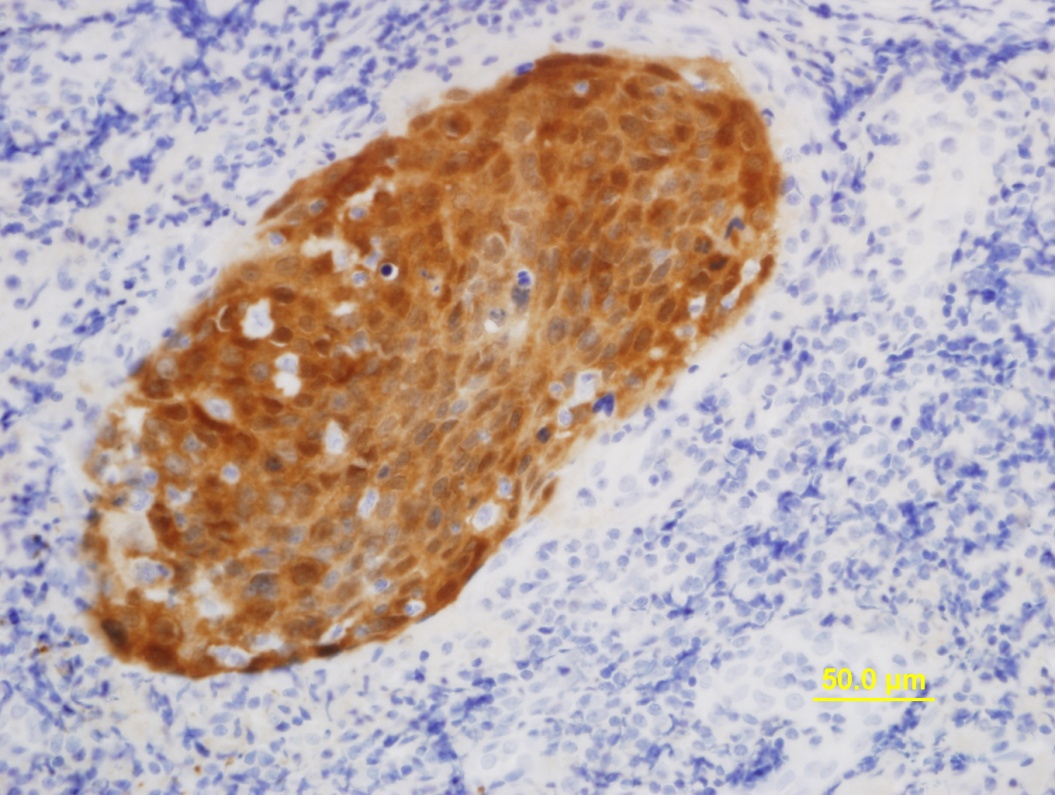


1. typical p16 staining for an HPV DNA-negative cancer


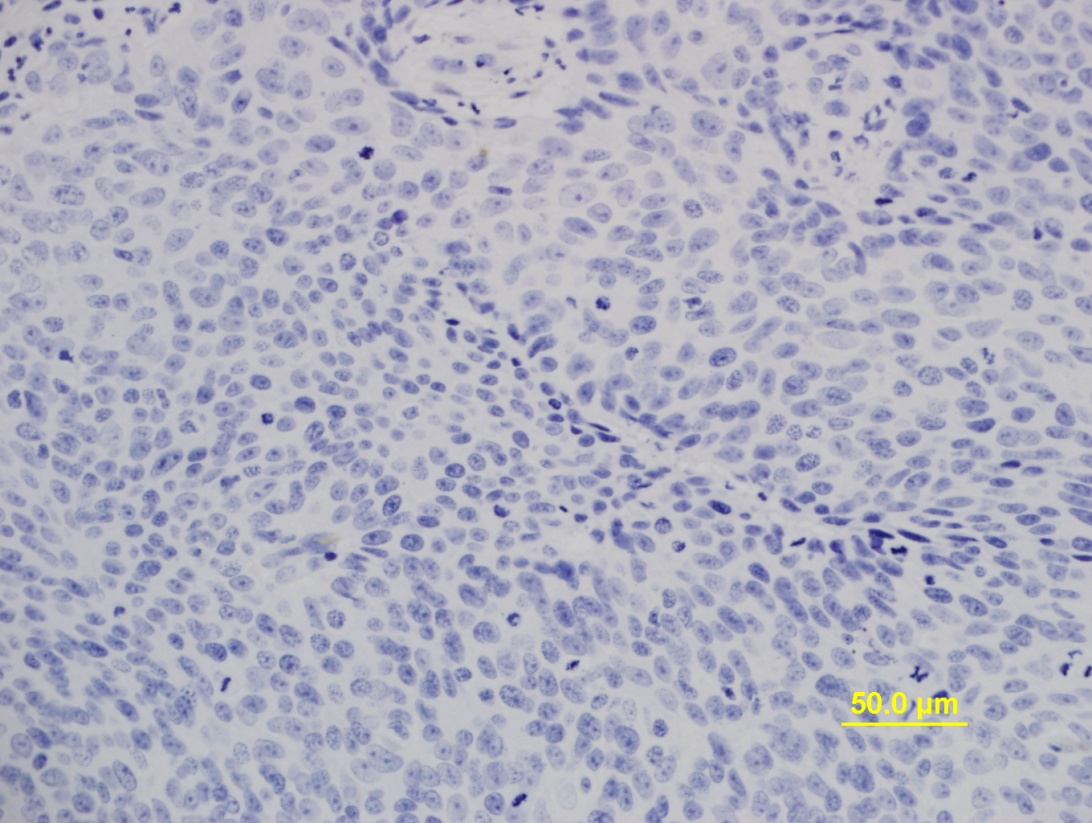


Supplementary Table 1

Relationship between HPV DNA and p16 from 198 tumor samples.

|  | HPV DNA-positive | HPV DNA-negative |
| --- | --- | --- |
| p16-positive | 83 | 3 |
| p16-negative | 21 | 91 |
| **Total** | **104** | **94** |
